# Spiritual Well-Being for Croatian Cancer Patients: Validation and Applicability of the Croatian Version of the EORTC QLQ-SWB32

**DOI:** 10.3390/ijerph182211920

**Published:** 2021-11-13

**Authors:** Ivana Dabo, Iva Skočilić, Bella Vivat, Ingrid Belac-Lovasić, Iva Sorta-Bilajac Turina

**Affiliations:** 1 Department of Epidemiology, Teaching Institute of Public Health of the Primorje—Gorski Kotar County, 51000 Rijeka, Croatia; 2 Department of Radiotherapy and Oncology, University Hospital Rijeka, 51000 Rijeka, Croatia; onkologija@kbc-rijeka.hr (I.S.); ingrid.belac.lovasic@medri.uniri.hr (I.B.-L.); 3 Marie Curie Palliative Care Research Department, Division of Psychiatry, Faculty of Brain Sciences, University College London, London W1T 7NF, UK; b.vivat@ucl.ac.uk; 4 Department of Oncology and Radiotherapy, University of Rijeka School of Medicine, 51000 Rijeka, Croatia; 5 Department of Social Medicine, Teaching Institute of Public Health of the Primorje—Gorski Kotar County, 51000 Rijeka, Croatia; iva.sorta-bilajac@zzjzpgz.hr; 6 Department of Environmental Medicine, University of Rijeka School of Medicine, 51000 Rijeka, Croatia

**Keywords:** cancer, EORTC, questionnaire, spiritual well-being, translation, validation

## Abstract

Spiritual well-being is a recognized predictor of health-related quality of life in palliative patients. No research in Croatia has yet addressed this field. This study, the first of its kind in Croatia, validated a Croatian translation of the EORTC QLQ-SWB32 measure of spiritual well-being with curative Croatian oncology patients and assessed its use and value. The study was conducted between July 2019 and January 2020 at the Department of Radiotherapy and Oncology, University Hospital Rijeka, with 143 cancer patients, using the linguistically validated Croatian version of the measure. All patients found the measure acceptable. Confirmatory factor analysis aligned with the structure found in previous studies. Cronbach’s alpha confirmed internal consistency. Female participants scored higher on the RSG (Relationship with Someone or Something Greater), RG (Relationship with God), and EX (Existential) scales, and on Global-SWB. Patients with breast and gynecological tumors scored higher on RG. Older patients scored lower on RSG, RG and EX. Retirees and those with below-average incomes scored lower on EX. Participants who identified as having no religion scored lower on RSG. Stage I cancer patients scored higher on RG. The Croatian version of the EORTC QLQ-SWB32 is an acceptable, valid, and reliable measure of SWB for Croatian cancer patients.

## 1. Introduction

Health-related quality of life (HRQoL) in oncology addresses the effects of disease and treatment on individuals. It is considered multidimensional, including physical and psychosocial elements [1], with a focus on treating pain and other symptoms that may be physical, psychosocial, and/or spiritual [2]. The Quality of Life Group (QLG) of the European Organization for Research and Treatment of Cancer (EORTC) has established a modular approach for developing measures of HRQoL for cancer patients, which assess a range of issues relevant to HRQoL [3,4].

Spiritual well-being (SWB) is increasingly recognized as a significant predictor of HRQoL in patients living with a potentially life-limiting disease such as cancer [5]; it has been shown that SWB correlates with HRQoL for palliative patients [6,7,8]. SWB may protect against suicidal thoughts, hopelessness, and the desire for accelerated death in terminally ill patients, and so may be considered indispensable for holistic care [5], assisting with increased adaptation to a malignant disease diagnosis and its acceptance [6,7,9]. However, no research in this area has previously been conducted in Croatia.

Members of the EORTC QLG recently completed international validation of a stand-alone functional measure of SWB, the EORTC QLQ-SWB32 (SWB32) [8]. This study was conducted cross-culturally with collaborators from 14 countries on four continents, in 10 languages, and with 451 participants receiving palliative care for cancer. The international and intercultural aspects helped to assure that the measure was suitable in participating cultures and contexts, and understandable and consistent in all tested languages [8,10].

This international study highlighted the importance of simultaneously addressing spiritual issues and needs alongside other important issues for patients receiving palliative care, thus complementing the holistic approach [8,11]. The SWB32 has been recognized as a means of intervening and beginning to address respondents’ spiritual problems and needs [8,10].

Amongst other recommendations, Vivat et al. [8] recommended exploring the use of the SWB32 with curative oncology patients. No such research has yet been conducted in Croatia, but two recent studies in China included curative patients [12,13] (it must however be noted that both these Chinese studies used a translation of the SWB32 which has not been validated by the EORTC Translation Unit). Following an initial small-scale study validating the Croatian translation of the SWB32, the primary aim of our project’s main study was to evaluate and validate the psychometric characteristics of the Croatian version of the SWB32 with Croatian curative oncology patients, and assess its suitability as both a measurement and intervention tool for these patients. The secondary aim was to conduct known-group comparisons, exploring whether there were any relationships between participants’ socio-demographic and medical characteristics and their responses to the SWB32. Lastly, we compared our findings with those from the international validation study and the other studies which have used a version of the SWB32 with curative patients.

## 2. Methods

### 2.1. Measure: EORTC QLQ-SWB32 (SWB32)

The SWB32 has 32 items, with 22 items forming four multi-item scales: Relationships with Others (RO) (six items); Relationship with Self (RS) (five items); Relationship with Someone or Something Greater (RSG) (five items); and Existential (EX) (six items); and a single item Relationship with God (RG) [8]. The measure also includes a Global-SWB item and nine other items: two items that screen for current or past belief in someone or something greater; three items that are only answered by those responding positively to the screening items; and four non-scoring clinically relevant items [8].

Global-SWB scores range from 1 (*very poor*) to 7 (*excellent*) plus the additional option of “0” for *I do not know* or *I cannot answer*. The other 31 items are scored from 1 (*not at all*) to 4 (*very much*). Sum scores from the four QLQ-SWB32 scales, the RG item, and the Global-SWB item are transformed into scores from 0 to 100, with 100 as the best possible score [8,10].

### 2.2. Patient Recruitment and Data Collection

The study was conducted at the Department of Radiotherapy and Oncology of the University Hospital Rijeka (UHR). UHR is one of the five university clinical hospital centres in Croatia. It is a regional referral centre for three counties (Primorje-Gorski Kotar, Istria and Lika-Senj), which provides medical care for about 600,000 inhabitants of Croatia (15%) [14]. The UHR Ethics Committee approved the study.

The SWB32 was translated from English to Croatian following the EORTC translation guidelines [15], including the EORTC standard translation procedure of two forward-back translations, Initial linguistic validation, i.e., assessment of the comprehensibility of the translated SWB32, was then performed in April 2019 with Department patients attending for oncology treatment. Subsequently, the Croatian version was approved by the EORTC QLG as the official Croatian version of the EORTC QLQ-SWB32.

For the larger main study, investigating the structure of the measure, a sufficient sample size was estimated by Power Analysis for ANOVA as N = 143, assuming a significance threshold of α = 0.05 and a test power of 0.8, calculated using G* Power.

The research team developed a Background Information Survey to collect socio-demographic characteristics: age, place of residence, education, employment, the economics of daily living, living conditions, marital status, distance from the health facility, and whether participants had religious beliefs or were part of any spiritual movement/organization. The medical data collected were: primary tumour site, cancer clinical stage (I–III), and the type of oncology treatment according to their medical history.

Patient participants were recruited between July 2019 and January 2020 from ambulatory oncology, radiotherapy, and daily hospital chemotherapy. Inclusion criteria were: aged between 30 and 65 years of age, diagnosed with stage I-III cancer for up to five years, receiving oncological treatment (neoadjuvant therapy and adjuvant therapy) at the department, able to read and write Croatian, and having provided informed consent. The lower age limit was chosen to reduce heterogeneity, since cancer is rare for people under 30 in this country; the upper age limit in order to reduce the likelihood of comorbidities which might confound the analysis. Exclusion criteria were: patients with metastases to distant organs, anyone with a psychotic mental disorder, unable to follow the instructions from the study, unable to read and speak Croatian fluently, and anyone who withheld consent.

Participants were generally approached one month after receiving their diagnoses, i.e., after they had started their oncology therapy and become familiar with their treatment plan. This timing was chosen because at this point patients would be more accustomed to their treatment, and less uncertain and fearful about it, while likely to be experiencing fewer strong adverse effects, and therefore more willing to be approached.

Authorized members of the research team delivered information about the study, which the participants could also read in detail on the participant information sheet. After informed consent was obtained, the researchers collected demographic data and provided participants with the SWB32 to complete on their own. Participants completed the questionnaires and the subsequent debriefing interviews while waiting for their scheduled oncological treatment.

While completing the measure, most participants requested an explanation for item 31 (“I have spiritual wellbeing”), because the phrase was unfamiliar, and participants associated SWB with religion. Researchers offered a brief explanation, following Frankl [16]: “search for the goal and meaning of life”.

After completion, researchers conducted face-to-face debriefing interviews with the participants. Previous research with the SWB32 has highlighted that the measure has an unavoidable interventionist character, since the act of completing it encourages reflection, and enables new insights into one’s condition and illness, thus prompting engagement in discussion and individual conversation [8,10]. The developers recommend, therefore, that a researcher and/or care professional should always be available for subsequent discussion if required [8,10]. Consequently, administering the SWB32 requires that research team members and/or other care professionals (mental health and/or spiritual care professionals) be present to offer emotional, psychological, or spiritual support if required after respondents have completed the measure. Accordingly, qualified members of the research team were present and available to all participants who might wish further support.

### 2.3. Statistical Analysis

Response data were summarized using basic statistical indicators (i.e., means, medians, ranges, and standard deviations). Confirmatory factor analysis and Cronbach’s alpha reliability estimate were used to validate the Croatian version of the measure. The threshold for Cronbach’s alpha was set at 0.6 [17]. None of the analyzed variables showed normality, and non-parametric methods were used in all statistical analyses. Specifically, the Mann–Whitney U-test was used to test differences between any two groups, and the Kruskal–Wallis test to test differences between more than two groups. Statistical analysis of the collected data was performed using the software Statistical Package for Medcalc 11.2.0.0 [18], and statistical significance of the tests was set at *p* < 0.05.

## 3. Results

### 3.1. Linguistic Validation

Fifteen patients participated in the linguistic validation in April 2019. Their data were used only for linguistic validation, and not the main study. These participants were all able to complete the measure independently and needed no additional clarification of the instructions. Three (#4, #10, #12) said that they liked the content and how the items were formulated. Two (# 5, #11) stated that the measure was clear and understandable, not requiring unnecessary interventions. Four (#1, #3, #6, #13) found item 27 (“I feel that I will live on through my words, deeds and/or influence on other people”) distressing, feeling that it explicitly raised their illness and death. Item 31 (“I have spiritual wellbeing”) required explaining to all participants, who associated SWB with religion.

### 3.2. Main Study Participants’ Socio-Demographic and Clinical Characteristics

A further 143 participants took part in the larger main study between July 2019 and January 2020. Just over half of these participants were women, 75 F (52.44%), and their median age was 58 years (range 32–65). Most participants completed the SWB32 in 15–20 min. No participants required additional support after discussing their responses with the researchers.

Most participants had completed high school (65.73%), and assessed their economic status as average (76.92%). They were most often married (67.83%), and most were Catholic (48.95%). Just over half (50.35%) were in permanent employment. Most had stage-II (41.26%) or stage-III disease (39.86%), and the largest patient group had gastrointestinal cancer (35.67%), followed by gynecological cancer (20.28%). Details of participants’ socio-demographic and clinical characteristics are shown in Table 1.

### 3.3. Factor Analysis and Reliability Analysis

Confirmatory factor analysis examined the structure of the Croatian SWB32 when used with curative patients. We compared this with findings from the international study with palliative patients [8,19]. Our study identified four factors with eigenvalues greater than 1 (Keiser criterion), explaining 66% of the variance. This result agreed with the number of factors found in the international validation study, and broadly with their content [8]. In order to determine the factor loads, a varimax rotation of the factor load matrix was performed. Almost all items were allocated to the same scales as in the international study [8], except for two items, which each loaded to two scales. Item 12 (“Able to forgive others”) was allocated to the RSG scale, rather than RO, as in the international study, although it loaded to both scales, and the difference in factor loads between the two was small (0.1339 (RO); 0.2567 (RSG)). The second difference appeared for item 31 (“I have spiritual well-being”), which was associated with EX instead of RSG, but also loading to both scales, with a minimal difference in the factor load (0.5718 (EX); 0.5171 (RSG)). The results are presented in Table 2.

Table 3 shows the reliability analysis of participant scores for each of the measure’s scales. We assessed the internal consistency of the measure using Cronbach’s alpha, and for all scales the value was satisfactory. For two scales (RSG and EX), Cronbach’s alpha was over 0.7, and for a third, RO, just under, at 0.68. Cronbach’s alpha for the fourth scale, RS, was slightly lower, at 0.62.

### 3.4. Known-Group Comparisons: Associations between Participants’ Scores on the SWB32 Scales and Their Socio-Demographic Characteristics and Clinical Parameters

Table 4 presents the comparisons for the known groups of our participants, with the associations between participants’ scores on the SWB32 scales, and their socio-demographic characteristics and clinical parameters.

We found statistically significant differences for some of the known-group comparisons. For sex, women’s scores for RSG, EX, and RG, and for Global-SWB were significantly higher than men’s. Relatedly, for type of cancer, there was a statistically significant difference for RG, with the post hoc analysis showing that people with breast and gynecological tumors (all women, other than one man with breast cancer) scored highest on this scale. The lowest scores on the RG scale were for people with urogenital tumors, who were all men. For disease stage, there were statistically significant differences for RG, where people with Stage I disease differed significantly, scoring higher than those in Stage II or Stage III.

There were statistically significant differences for age for RSG, EX, and RG. Post hoc analysis for EX indicated a clear difference between people younger or older than 50, with lower scores for older participants. For RG and RSG, scores for participants younger than 50 were statistically significantly different from the other two groups, with younger participants scoring higher than older.

For employment status, EX scores were different, with post hoc analysis finding a statistically significant difference between retirees and permanent employees. In contrast, unemployed/temporary employees did not differ from the other two groups. Descriptively, retirees showed lower values on this scale. For economic status, only EX scores were statistically significantly different, with participants with below-average incomes differing from the other two groups, with lower scores. For religious affiliation there was a statistically significant difference only for RSG, with non-believers having statistically significantly lower values compared to the other two groups. For education, marital status, and type of treatment, no statistically significant differences were found on any scale.

## 4. Discussion

Vivat et al. [8] state that the EORTC QLQ-SWB32 measure offers possibilities for future research and clinical practice in a field where there is currently no “gold standard” [8,20]. Our study engages with this observation and contributes to further understanding of the multiple facets of SWB, and its place and role in different clinical and cultural settings, providing additional insight by investigating the use of the measure with Croatian curative oncology patients of both sexes, and with a variety of cancers. As Feng et al. [13] point out, understanding the role of spirituality and SWB for both curative and palliative patients is important in developing and delivering holistic and culturally appropriate patient-oriented care.

The primary aim of our main study was to explore the structure and validity of a Croatian version of the SWB32 with patient participants in Croatia. We found that this measure was comprehensible and acceptable to all participants, with quantitative findings broadly equivalent to those found in the international study with palliative patients [8], Median values of scale scores in our study were mostly consistent with those in other published studies [8,12,13,19]. For each of the four multi-item scales, the Cronbach’s alpha coefficient was above the threshold of 0.6.

Vivat et al. [8,10] have pointed out that the SWB32 is also an intervention tool because it opens space for additional insights into patients’ experiences. Our linguistic validation also found this, with participants generally satisfied with the content of the SWB32 and the way its items are conceived. They felt that through their interaction with the tool they had gained deeper insights into themselves and ways of coping with the disease, and they recognized the connection between SWB and their own QoL. They thought that the SWB32 would be a useful tool in oncology treatment, and that it was important to have members of the research team or other health care professionals available after completing it.

Four of the 15 participants found item 27: “I feel that I will live on through my words, deeds and/or influence on other people” upsetting. Conversations with these participants found that this was because this item directly confronted them with their illness and fear of death, an issue with which palliative patients are already dealing, but patients at early stages of disease may not yet have faced directly. A cancer diagnosis and its subsequent treatment often create a crisis for patients [21,22,23,24], as they are confronted with the loss of bodily functions, emotional anguish, deteriorating physical conditions, and the threat of death [25,26,27,28,29]. In one study [27], participants expressed considerable fear of recurrence despite their disease-free interval, indicating that even four years after diagnosis, cancer and primary treatment still have an effect on even curative patients’ lives, through fear and the need for emotional coping.

The chief difference between quantitative findings from our main study and those from the international validation study [8] was a variation in the factor loads for two items (item 12 and item 31), which each grouped in a different scale from the international study. However, each item also grouped slightly less strongly in a second scale, and, for each, this second scale was the same as that in which they grouped in the international study.

In our study, item 12 (“Able to forgive others”) grouped most strongly in RSG, but also, although slightly less strongly, in RO, as in the international validation [8]. The concept of forgiveness has deep roots in religion across faiths and cultures [30,31]. It has been shown to partially mediate the relationship between religion and health [32]. The practice of religion and spirituality is one of the positive indicators of mental and spiritual well-being [12,32], and thus relates conceptually both to a relationship with someone or something greater (RSG) and to relationships with other people (RO).

Item 31 (“I have spiritual well-being”) grouped most strongly in EX in our study, but also, although slightly less strongly, in RSG, as in the international validation [8]. The need to explain the phrase (“spiritual well-being”) occurred repeatedly with our participants. Most associated SWB with religion, and so became “stuck” on the item, as the linguistic validation also found. The brief explanation offered, Frankl’s “search for the goal and meaning of life”, [16] may have affected our participants’ responses, and hence also affected the factor loading for that item.

The secondary aim of our study was to investigate known-group comparisons, examining associations between participants’ socio-demographic characteristics and clinical parameters and their scores on the SWB32 scales. We found statistically significant differences for several of our study variables, but, like the international SWB32 validation study [19], we found no differences for marital status, in contrast to other studies with other SWB measures [33,34], although it should be noted that the SWB32 and these measures have distinct conceptualisations of SWB [20], and that these studies were conducted in the USA, so their findings may reflect specific geo-cultural effects.

For **sex**, women scored higher on RSG, EX, RG, and Global-SWB. In the validation study [8,19], and the two Chinese studies [12,13], female participants also scored significantly higher on RSG, EX, and Global-SWB. Studies using other measures of spirituality or SWB (the Spiritual Interests Related to Illness Tool (SpIRIT) [33] and the Spiritual Needs Inventory (SNI) [35]) have also found that women have greater SWB than men [33,34,35,36]. The only statistically significant difference for **types of cancer** we found was for RG scores, with higher scores from participants with breast and gynecological tumors, all but one of whom were women (one man had breast cancer). The lowest RG scores were from patients with urogenital tumors, all of whom were men. It is important to note also that, while some studies have found that women with gynecological tumours may report worse HRQoL [28,29], women are more likely than men to utilize cancer information services and other support services [37,38].

For **disease stage**, we found statistically significant differences for the RG scale, with Stage I patients scoring higher than those in Stages II or III. Our curative participants had higher scores on every scale in the SWB32 than patients with advanced cancers, similarly to Chen et al.’s study with patients with primary gynecological cancer [12]. As Chen et al. [12] recommend, future studies should compare the SWB scale scores for patients in different cancer stages.

For **age**, we found no statistical difference for older age on RS, and negatively associated scores for all other scales, with EX scores being lowest for the oldest participants. RG and RSG scores for respondents under 50 were statistically significantly different from older respondents, and these younger respondents’ scores for Global-SWB were descriptively higher. Our older participants found items directly pertaining to religion and spirituality particularly difficult. Rohde et al. [19], however, found that RS scores were positively associated with older age; and other studies using other measurement tools have also found weak positive associations of SWB with increasing age [35,39]. The difference for our study could be because Croatia was a communist country for fifty years, where religious practice was officially forbidden, thus performed in secrecy. Croatia became a secular state in the 1990s, so adherence to religion may be understood as a relatively novel concept in our society, and therefore more acceptable to people under 50 years of age [40].

For **employment** and **economic status**, we found a statistically significant difference between retirees and permanent employees, i.e., participants with below-average incomes in relation to other groups on EX. Retirees and those with below-average incomes had lower scores. The burden of existential issues in these groups can be explained by data from the Croatian Bureau of Statistics [41]. In 2019 in Croatia, the at-risk-of-poverty rate was 18.3%, and this was among people aged 65 or over (30.1%), especially women (33.6%). The at-risk-of-poverty rate was also highest for the unemployed, at 45.3% [41]. Such patients find it difficult to make plans for the future, engage in activities they love, feel fulfilled, or cope well with problems.

For **religious affiliation**, we found a statistically significant difference for RSG, with statistically significantly lower values for people who indicated in their responses to our Background Information Survey that they were not religious. In Chen et al. [12], patients with formal religious affiliations had higher scores on the EX and RSG scales. In Feng et al. [13], where 90% of participants had no religious belief, the lowest scores were for RSG.

However, it is not always simple to identify what individuals believe [8]. Some people may say that they have no religious or spiritual beliefs, and have never had them, but may still indicate elsewhere that they have some such beliefs [8]. The SWB32 international validation study found this for 73 of the 156 respondents who had stated that they had no religion nor involvement with any spiritual movement [8]. Similarly, while 42.7% of our participants responded to our Background Information Survey that they had no religious beliefs, the majority of these “not-religious” participants’ responses to items 22 and 23 nevertheless indicated that they believed in someone or something greater. Thus, these respondents should not be considered as pure atheists. This suggests that dividing “not-religious” into atheists and agnostics might be helpful for further research using the SWB32 in Croatia, where, in the most recent census [42], 86.3% of the population identified as Christian and Catholic.

The SWB32 was designed to be suitable for people with various religious faiths or spiritual beliefs, and those with none [8]. Astrow et al. [43] found that patients who did not subscribe to any specific religion had greater spiritual needs than those who did. This suggests that health care providers should also attend to spiritual care for patients who say that they have no religion, and perhaps provide more of this kind of care, or combine it with psychological counselling [12].

We also compared our participants’ SWB32 scores with those of participants in other studies. Our study participants were all curative patients with clinical stages I-III of the disease, without metastases, whereas other research studies have included palliative patients and those with advanced cancer, including metastases to distant organs [6,12,19]. Our participants’ Global-SWB scores were similar to those in the further analysis of the data from the international validation study with palliative patients [19], but our participants’ scores for RS and EX were higher than in that study. We had hypothesized that this might be the case for RS, since curative oncology patients may be more focused on everyday situations and specific life concepts. Two Chinese studies using the SWB32 included curative patients [12,13], and both found that these patients scored higher than palliative patients on EX, RS, and Global-SWB. However, since both these studies used a Chinese translation of the SWB32 which the EORTC Translation Unit has not yet validated, it is unclear how that version relates conceptually to EORTC-validated versions, so any comparison of results must be treated with caution.

## 5. Study Strengths and Limitations

We administered the SWB32 to a sample of roughly equal proportions of men and women. Our study kept a clear focus on curative cancer patients, strictly following the inclusion criteria to reduce confounding variables from advanced cancer stages, or other diseases with distinct trajectories, possible complications and comorbidities, and treatments, which could impact SWB. As recommended in previous studies using the SWB32, we introduced economic status variables and found statistically significant differences. Our quantitative comparison found that our study and findings are consistent and comparable with other studies using the SWB32, although our approach varied slightly from that of the international validation.

The validation of the instrument was performed for the Croatian language, but the Croatian version of the SWB32 could perhaps also be used in neighboring countries, where the Croatian language is understood and used both in oral and written communication.

The majority of our participants requested an explanation of SWB, and we offered Frankl’s “search for goal and meaning” [16]. This may have affected participants’ responses, thereby causing the minimal differences for two items in our factor analysis, compared to the international validation study [8]. However, our sample size was considerably smaller than that of the international validation (143 participants rather than 451), and far less culturally diverse (respondents from a single country and using one language, rather than fourteen countries and ten languages). This may have influenced the factor analysis, and also the scale reliability.

Our study sample was recruited in one of the five Croatian university clinical hospital centres. It therefore has some relationship with the situation in the general population. However, generalising our results to all cancer patients in Croatia is not advisable, since there are some religious and cultural differences between coastal and continental Croatia. Investigating these might be a direction for future research using the SWB32.

## 6. Conclusions

In conclusion, our linguistic validation confirmed the interventional character of the EORTC QLQ-SWB32, and the confirmatory factor analysis for the main study showed that the measure retained broadly the same structure for Croatian curative patients as for the palliative patient participants in the international validation. Cronbach’s alpha confirmed the internal consistency of the measure, with values above the satisfactory threshold.

Known-group comparisons found statistically significant differences for participants’ scores on some scales for sex, age, cancer stage, and religious beliefs. We also found some descriptive differences between our participants for some variables. Future larger-scale research studies might investigate these potentially relevant variables in more depth, with special emphasis on age, sex, and religion, and further differentiation for the latter between believers, agnostics, and atheists. Studies might also seek to identify reference intervals for sex and age groups.

The Croatian validated version of the QLQ-SWB32 is a valid and reliable instrument for measuring SWB for curative cancer patients in Croatia. As with other validated versions, this measure is available for use in research and clinical practice, as both a measurement and intervention tool. In clinical practice, the measure could be used to identify those curative cancer patients who are experiencing lower SWB, have unmet needs, and, therefore, need more customized spiritual care as an integral part of holistic, person-centred healthcare, as well as being used to initiate those spiritual care interventions, through prompting discussion. Treatment protocols might be modified and extended to integrate assessments of patients’ spiritual wellbeing, including strategies and approaches for assisting those with lower scores.

## Figures and Tables

**Table 1:** Main study participants’ demographic and clinical characteristics.

	*N* (Total = 143)	%
**Age**		
30–49	39	27.27
50–60	59	41.26
>60	45	31.47
**Sex**		
Male	68	47.55
Female	75	52.44
**Education level**		
Illiterate	1	0.70
Elementary school	11	7.69
Craft	3	2.10
High-school	94	65.73
Higher Education	10	6.99
University	23	16.08
Master, Doctorate	1	0.70
**Employment status**		
Unemployed	15	10.49
Temporary employment	10	6.99
Permanent employment	72	50.35
Retired	42	29.37
Other	4	2.80
**Economic status**		
Below average	25	17.48
Average	110	76.92
Above average	8	5.59
**Marital status**		
Married	97	67.83
Divorced	18	12.59
Single	14	9.79
Other	14	9.79
**Religion**		
Not religious	61	42.66
Catholic	70	48.95
Orthodox	2	1.40
Muslim	7	4.90
Jehovah’s Witness	1	0.70
Other	2	1.40
**Cancer clinical stage**		
I	24	16.78
II	59	41.26
III	57	39.86
Unknown	3	2.10
**Cancer site**		
Breast	26	18.19
Gastro-intestinal	51	35.67
Urogenital	19	13.27
Gynecological	29	20.28
Other	18	12.59
**Type of oncology treatment**		
Radiotherapy	34	23.78
Chemotherapy	74	51.75
Hormonal treatment	4	2.80
Surgical treatment	11	7.69
Chemotherapy + Radiotherapy	13	9.09
Hormonal treatment + Radiotherapy	6	4.20
Unknown	1	0.70

**Table 2:** Factor load.

	EX	RO	RSG	RS
**Eigenvalue**	5.19058	1.99884	1.27951	1.08595
**Cumulative variance**	0.3586	0.4967	0.5850	0.6601
Q1 Able to deal with problems Moći se nositi s problemima	**0.6200**	0.0801	−0.0279	0.5387
Q2 Peace with myself Osjećaj mira sa samim sobom	**0.3127**	0.2015	0.0386	−0.4878
Q3 Find things I enjoy Moći pronaći stvari u kojima uživam	**0.5195**	0.1164	0.1557	−0.2503
Q5 Troubled Osjećaj uznemirenosti	−0.2424	−0.2022	0.0440	**0.4745**
Q6 Lonely Osjećaj usamljenosti	−0.2524	−0.3816	0.0793	**0.4298**
Q8 Share thoughts with those close to me Moći podjeliti misli o životu s osobama koje su mi bliske	0.3047	**0.4083**	0.2769	−0.1041
Q9 Loved by those important to me Osjećati se voljeno od strane onih koji su mi važni	0.0882	**0.5872**	0.0480	−0.0863
Q10 Someone to talk about my feelings Imati nekog s kim mogu razgovarati o mojim osjećajima	0.1613	**0.6343**	0.1633	−0.0866
Q11 Able to trust others Imati povjerenje u druge	0.0380	**0.4849**	0.1427	−0.1579
Q12 Able to forgive others Moći oprostiti drugima	−0.0175	**0.1339**	**0.2567**	−0.1988
Q13 Valued as a person Osjećati se vrijedim kao osoba	0.4010	**0.4248**	0.0803	−0.1319
Q14 My life is fulfilling Osjećaj da je moj život ispunjen	**0.4661**	0.3829	0.1645	−0.3461
Q15 My life is worthwhile Osjećaj da je moj život vrijedan življenja	**0.5368**	0.4375	0.1207	0.0382
Q16 Plan for the future Moći stvarati planove za budućnost	**0.5580**	0.3599	−0.0327	0.0513
Q17 Worries/concerns about the future Brige/zabrinutosti za budućnost	−0.0840	−0.0824	−0.0139	**0.5296**
Q18 Can anything be done for me Pitao/la sam se može li se išta za mene učiniti	−0.1162	−0.0988	0.0190	**0.4707**
Q19 Unfair that I am ill Osjećam da je nepravedno što sam bolestan/a	0.0052	0.1314	0.0236	**0.5001**
Q20 Time for quietness/prayer/meditation Vrijeme za tišinu/molitvu/meditaciju	0.0719	0.1624	**0.4667**	−0.0300
Q21 Important that others pray for me Osjećam da je važno da drugi mole za mene	−0.0084	0.1078	**0.7488**	0.0652
Q27 Live on through words, deeds... Živjeti kroz riječi, djela...	0.4188	0.2177	**0.4653**	0.0197
Q30 I believe in life after death Vjerujem u život poslije smrti	0.0952	0.0656	**0.6968**	−0.0156
Q31 I have spiritual well-being Ispunjen/a sam duhovnim blagostanjem	**0.5718**	−0.1124	**0.5171**	−0.1309

Note: bold text indicates the largest factor loadings for each item.

**Table 3:** Analysis of scale reliability.

Scale	Number of Items in Scale	Cronbach’s Alpha	Median	Range
Relationship with Others (RO)	6	0.68	72.22	22.22–100.00
Relationship with Self (RS)	5	0.62	73.33	20.00–100.00
Relationship with Someone or Something Greater (RSG)	5	0.75	60.00	6.67–100.00
Existential (EX)	6	0.76	72.22	5.56–100.00
Relationship with God (RG)	1	-	33.33	0.00–100.00
Global-SWB	1	-	66.67	16.67–100.00

**Table 4:** Known-group comparisons: Associations between participants’ scores on the SWB32 scales and participants’ socio-demographic characteristics and clinical parameters.

**Socio-Demographic and Clinical Characteristics**	**Scale** **Median (Range)**					
	**RO**	**RS**	**RSG**	**EX**	**RG**	**Global-SWB**
**Sex**						
Male	72.22 (22.22–100.00)	73.33 (20.00–100.00)	53.33 (6.67–100.00)	72.22 (5.56–100.00)	33.33 (0.00–100.00)	66.67 (16.67–100.00)
Female	72.22 (38.88–100.00)	73.33 (20.00–100.00)	60.00 (13.33–100.00)	72.22 (38.89–100.00	66.66 (0.00–100.00)	83.33 (33.33–100.00)
*p* **	0.631	0.589	0.033	0.045	0.000	0.001
**Age**						
<50	72.22 (55.56–100.00)	66.67 (20.00–100.00)	60.00 (26.67–100.00)	77.78 (44.44–100.00)	66.67 (0.00–100)	83.33 (50.00–100.00)
50–60	72.22 (22.22–100.00)	73.33 (20.00–100.00)	53.33 (6.67–93.33)	72.22 (5.56–100.00)	33.33 (0.00–100.00)	66.67 (16.67–100.00)
>60	72.22 (38.89–100.00)	73.33 (26.67–100.00)	53.33 (6.67–93.33)	66.67 (38.89–100.00)	33.33 (0.00–100.00)	66.67 (16.67–100.00)
*p* *	0.073	0.129	0.014	0.001	0.011	0.087
**Education**						
No education, ES, and profession	72.22 (50.00–94.44)	73.33 (26.67–93.33)	46.67 (20.00–93.33)	61.111 (44.44–100.00)	50.00 (0.00–100.00)	66.67 (33.33–100.00)
High school	72.22 (22.22–100.00)	73.33 (20.00–100.00)	60.00 (6.67–100.00)	72.222, (5.56–100.00)	33.33 (0.00–100.00)	66.67 (16.67–100.00)
University and higher	75.00 (38.89–94.44)	76.67 (20.00–100.00)	56.67 (6.67–100.00)	72.22 (38.89–100.00)	66.67 (0.00–100.00)	83.33 (33.33–100.00)
*p* *	0.714	0.786	0.651	0.236	0.099	0.233
**Employment status**						
Unemployed Temporary employment	72.22 (38.89–100.00)	73.33 (46.67–100.00)	60.00 (26.67–100)	72.22 (44.44–94.44)	33.33 (00–100.00)	66.67 (33.33–100.00)
Permanent employment	72.22 (38.89–100.00)	73.33 (20.00–100.00)	53.33 (6.67–100.00)	72.22 (38.89–100.00)	50.00 (0.00–100.00)	75.00 (16.67–100.00)
Retiree/other	72.22 (22.22–100.00)	66.67 (20.00–100.00)	63.33 (6.67–93.33)	66.67 (5.56–100.00)	66.67 (0.00–100.00)	66.67 (16.67–100.00)
*p* *	0.109	0.556	0.744	0.021	0.545	0.132
**Economic status**						
Below average	72.22 (22.22–100.00)	66.67 (20.00–100.00)	60.00 (13.33–93.33)	66.67 (5.56–88.89)	33.33 (0.00–100.00)	66.67 (16.67–100.00)
Average	72.22 (38.89–100.00)	73.33 (20.00–100.00)	60.00 (6.67–100.00)	72.22 (38.89–100.00)	66.67 (0.00–100.00)	66.67 (16.67–100.00)
Above average	72.22 (38.89–88.89)	86.67 (53.33–93.33)	46.67 (20.00–60.00)	77.77 (50.00–100.00)	33.33 (0.00–100.00)	83.33 (33.33–100.00)
*p* *	0.103	0.160	0.168	0.030	0.466	0.322
**Marital status**						
Married	72.22 (38.89–100.00)	73.33 (20.00–100.00)	60.00 (6.67–100.00)	72.22 (38.89–100.00)	66.67 (0.00–100.00)	67.00 (16.67–100.00)
Divorced	66.67 (38.89–100.00)	70.00 (40.00–100.00)	56.67 (20.00–93.33)	6.67 (38.89–100.00)	3.33 (0.00–100.00)	67.00 (17.00–100.00)
Single	77.78 (22.22–88.89)	76.67 (20.00–100.00)	56.67 (20.00–100.00)	75.00 (5.56–100.00)	33.33 (0.00–100.00)	83.00 (50.00–100.00)
Other	77.78 (38.89–94.44)	76.67 (20.00–100.00)	56.67 (13.33–93.33)	75.00 (38.89–100.00)	50.00 (0.00–100.00)	67.00 (16.67–100.00)
*p* *	0.291	0.610	0.987	0.417	0.260	0.340
**Religious affiliation**						
Not-religious	72.22 (38.89–100.00)	73.33 (20.00–100.00)	53.33 (6.67–100.00)	72.22 (38.89–100.00)	33.33 (0.00–100.00)	66.67 (16.67–100.00)
Catholic	72.22 (22.22–100.00)	73.33 (20.00–100.00)	60.00 (6.67–100.00)	72.22 (5.56–100.00)	66.67 (0.00–100.00)	66.67 (16.67–100.00)
Other	75.00 (55.56–100.00)	80.00 (53.33–86.67)	63.33 (46.67–93.33)	66.67 (50.00–88.89)	66.67 (0.00–100.00)	83.33 (16.67–100.00)
p*	0.960	0.516	0.008	0.479	0.075	0.115
**Disease stage**						
I	77.78 (44.44–100.00)	73.33 (40.00–100.00)	63.33 (26.67–100.00)	72.22 (44.44–100.00)	66.67 (0.00–100.00)	66.67 (50.00–100.00)
II	72.22 (22.22–100.00)	73.33 (20.00–100.00)	60.00 (6.67–93.33)	66.67 (5.56–100.00)	33.33 (0.00–100.00)	66.67 (16.67–100.00)
III	72.22 (38.89–100.00)	73.33 (20.00–100.00)	53.33 (6.67–100.00)	72.22 (38.89–100.00)	33.33 (0.00–100.00)	66.67 (16.67–100.00)
*p* *	0.627	0.433	0.177	0.082	0.018	0.664
**Type of cancer**						
Breast	77.78 (44.44–94.44)	73.33 (40.00–100.00)	60.00 (33.33–93.33)	77.78 (50.00–94.44)	66.67 (0.00–100.00)	83.00 (50.00–100.00)
Digestive system	72.22 (38.89–100.00)	73.33 (40.00–100.00)	60.00 (6.67–100.00)	72.22 (38.89–100.00	66.67 (0.00–100.00)	67.67 (33.33–100.00)
Urogenital system	77.78 (22.22–100.00)	80.00 (20.00–100.00)	53.33 (6.67–80.00)	72.22 (5.56–100.00)	33.33 (0.00 –66.67)	66.67 (16.67–100.00)
Gynecological System	72.22 (55.56–100.00)	73.33 (20.00–100.00)	60.00 (20.00–100.00)	72.22 (44.44–100.00)	66.67 (0.00–100.00)	67.00 (50.00–100.00)
Other	72.22 (38.89–100.00)	66.67 (20.00–100.00)	50.00 (13.33–100.00)	66.67 (38.89–100.00)	33.33 (0.00–66.67)	67.00 (16.67–100.00)
*p* *	0.980	0.705	0.140	0.186	0.007	0.462
**Type of oncological treatment**						
Radiotherapy	72.22 (22.22–100.00)	73.33 (20.00–100.00)	50.00 (13.33–93.33)	66.67 (5.56–100.00)	33.33 (0.00–100.00)	66.67 (16.67–100.00)
Chemotherapy	72.22 (38.89–100.00)	73.33 (20.00–100.00)	60.00 (6.67–100.00)	72.22 (38.89–100.00)	33.33 (0.00–100.00)	66.67 (33.33–100.00)
Combination /Other	72.22 (55.56–100.00)	73.33 (33.33–100.00)	60.00 (6.67–100.00)	72.22 (38.89–100.00)	66.67 (0.00–100.00)	66.67 (33.33–100.00)
*p* *	0.788	0.826	0.097	0.396	0.386	0.820

* Kruskal–Wallis, ** Mann–Whitney.

## Data Availability

The datasets used and/or analysed during the current study are available from the corresponding author on reasonable request.
